# Statements of Austrian hospices and palliative care units after the implementation of the law on assisted suicide: A qualitative study of web-based publications

**DOI:** 10.1007/s00508-023-02157-9

**Published:** 2023-03-09

**Authors:** Anna Kitta, Franziska Ecker, Elisabeth Lucia Zeilinger, Lea Kum, Feroniki Adamidis, Eva Katharina Masel

**Affiliations:** Clinical Division of Palliative Medicine, Department of Internal Medicine I, Medical University of Vienna, Waehringer Guertel 18–20, 1090 Vienna, Austria

**Keywords:** Physician-assisted suicide, Information science, Health communication, Religion and medicine, Autonomy

## Abstract

**Background:**

Since January 2022, assisted suicide (AS) in Austria is legal under certain conditions. One of these conditions is informative consultations with two physicians, one of whom must be qualified in palliative medicine. Patients who are thinking about AS can approach palliative care institutions. This study aims to assess the availability and nature of Austrian palliative care institutions’ web-based statements about AS.

**Methods:**

In this qualitative study, the websites of all Austrian palliative care units (*n* = 43) and all Austrian inpatient hospices (*n* = 14) were searched for possible statements on AS once in February 2022 and once in August 2022 using the three search terms “suicide”, “assisted”, and “euthanasia”. The findings were subsequently evaluated using thematic analysis and NVivo software.

**Results:**

Statements or texts that included positions on AS were found on the websites of 11 institutions (19%). The results covered three main themes 1) demarcation: denial of involvement and judgment about AS, 2) duty: handling of requests and describing the target group of care recipients, and 3) explanation: experience, values, concerns, and demands.

**Conclusion:**

The results of this study indicate that people in Austria who wish to have AS and who may use the internet as their first source of information largely find no relevant information. There is no online statement of a palliative care or hospice institution that endorses AS. Positions on AS are mostly lacking, while reluctant attitudes of Christian institutions are predominant.

**Supplementary Information:**

The online version of this article (10.1007/s00508-023-02157-9) contains supplementary material, which is available to authorized users.

## Introduction

In Austria, assisted suicide (AS) is regulated by the recently adopted federal Assisted Dying Act (Sterbeverfügungsgesetz, StVfG) Federal Law Gazette (Bundesgesetzblatt) BGBl. I Nr. 242/2021, which allows terminally ill people access to medically assisted under some conditions. The legislative change was initiated in 2019 by 4 applicants who filed a complaint with the Supreme Court for Constitutional Cases because they considered the ban on AS to be unconstitutional for several reasons. In December 2020, the Supreme Constitutional Court repealed the ban on AS and instructed the legislature to set up a law to regulate AS until the end of 2021. Additionally, experts, health care institutions, and the government were encouraged to specify policies concerning the new law. On 16 December 2021, the final version was adopted by the parliament, and in January 2022 the Assisted Dying Act (StVfG) was implemented. It is worth noting that the timeframe to set up the StVfG (1 year) and for the subsequent review of the first draft (3 weeks) was very short, and therefore experts and healthcare institutions had only a limited chance to engage in the process [1]. If the law had not been adopted before January 2022, AS would have been legal without any conditions or limitations.

Based on the current regulations, applicants for AS have to meet several conditions; they must be at least 18 years old, reside in or be a citizen of Austria, and be fully competent to make decisions. Furthermore, AS is only possible for people who suffer from a fatal disease or a severe long-term illness with persistent symptoms. People requesting AS must declare that their wish to die is voluntary and self-determined, and they have to carry out the last act themselves, for example, by drinking a lethal solution. A third party may help by, for example, picking up the lethal drug from a pharmacy (see Table 1). Two physicians, one of whom must be a specialist in palliative medicine, have to be consulted by the applicant and have to document the patient’s decision in the electronic Sterbeverfügungsregister (register of decrees) of death wills of the Federal Ministry for Social Affairs, Health, Care and Consumer Protection. Both physicians need to provide information about alternatives to AS, the dose of the lethal drug (sodium pentobarbital), concomitant medication, and possible side effects. Furthermore, the voluntariness and self-determination of the patient need to be assessed if the other conditions of the StVfG are met.

**Table 1:** Pathway of assisted suicide in Austria

**General preconditions**	– *≥ 18 years of age* – *Decision-making capacity must be proven* – *Austrian citizenship or residency in Austria* – *Disease (severe, permanent illness with persistent symptoms, or incurable illness)*
**Medical consultation**	***Two physicians need to educate patients about*** – *possible alternatives, in particular hospice care and palliative medical measures, as well as the possibility of drawing up a living will or other precautionary instruments, particularly having a health care proxy and precautionary dialogue* – *the dosage of the preparation and the concomitant medication necessary for the tolerability of the preparation* – *the method of taking the preparation, the effects and possible complications of taking the preparation, and that life-saving treatment can be refused with an advance directive* – *concrete offers for a psychotherapeutic consultation and for suicide prevention counselling, and* – *a reference to any other counselling services that may be useful in the specific case* ***One of the two physicians must*** – *have a diploma in palliative medicine* – *confirm the underlying disease*
**Sterbeverfügung (death warrant)**	Issued by a notary or patient advocate 12 weeks after the medical consultations at the earliest of 2 weeks in the case of very short life expectancy
**Drug**	Sodium pentobarbital (15 g) can be picked up at a pharmacy within 1 year
**Assisting person**	Provides assistance by picking up the drug from the pharmacy and/or by helping with the administration The person providing assistance is not allowed to be the same person providing the information or documenting the living will must not be notary, patient advocate must not be one of the two physicians who provided the required medical consultations

Following a mandatory period of 12 weeks after the second medical consultation (2 weeks in the case of terminal illness), the final documents must be approved by a notary or patient advocate. Subsequently, the lethal drug can be picked up from a public pharmacy within 1 year. The suicide itself is intended to be carried out in a private place [1].

However, within the first months after the StVfG came into force, an obstacle to the practical implementation of AS became evident. People desiring AS hardly ever found physicians offering the required consultations. Therefore, the question of what can be found on the internet arises when searching for AS in Austria. This qualitative study analyzed web-based statements about AS on the websites of Austrian palliative care units and hospices.

## Material and methods

### Data collection

This study used a qualitative study design, and data were initially collected in February 2022, a little more than 1 month after the new law came into force. A second round of data collection was conducted 6 months later in August 2022. At both times all available websites of Austrian palliative care units and hospices were accessed. To identify their internet presence, the Austrian umbrella organization for hospice and palliative care was used. The organization lists the institutions, addresses, and webpages ordered by the nine federal states [2]. Mobile palliative care teams and palliative consulting services were not included in the study. All websites of Austrian palliative care units (*n* = 43) and Austrian inpatient hospices (*n* = 14) were searched for possible statements about AS using the following three search terms: “suicide” (German: Suizid), “assisted” (German: assistiert), and “euthanasia” (German: Sterbehilfe).

Using the abovementioned search terms, linked interviews, articles, and short notes on the topic of AS were found that did not have the nature of a formal statement; however, as they underlined a certain position of the palliative care units and hospices, they were included in the analysis and were treated as forms of indirect information.

### Data analysis

Data were analyzed using qualitative thematic analysis [3–6]. A set of codes was generated via data familiarization and a thorough reading of the different statements and information published on the websites. The coding process started while reading the first statements and was continued throughout the entire analysis process. Data were analyzed using the software NVIVO (QSR International Pty Ltd., Melbourne, Australia). Three researchers (AK, FE, and ELZ) independently generated a list of codes inductively out of four different web-based statements. The results were then compared. There was a high level of agreement between the encoders, and minor differences were resolved by group discussion with three other researchers (LK, FA, EKM).

The codes provided structures of order and served as indicators of relevant details about the statements. The systemization, comparison, and generation of themes from coded and collated data followed (see Table 2). Themes were identified, reviewed, defined, refined, and named by consensus among the team members. Citations in the present report were translated by a professional translator.

**Table 2:** Overview of the analysis structure

Themes	Categories	Codes
*Demarcation*	Denial of involvement	No provision of help
		Not the place for assisted suicide
	Judgment about assisted suicide	Devaluation
		Interpretations, “cry for help”
		Word choice
		Pictorial language
*Duty*	Handling of the request	Take it seriously
		Search for alternatives
		Self-determination
	Target group	Nobody left alone
		Caregiver
		Employees
*Explanation*	Experience	Observations
		Perceived knowledge
	Values	Protection of life
		Christian
		Embeddedness
	Concerns and demands	Financial concerns
		Concern about employees
		Responsibility of society
		“slippery slope”, “Pandora’s box”

## Results

### Qualitative findings

There are 43 palliative care units in Austria; of these, 9 are run by Christian organizations and the rest are publicly funded. Additionally, there are 14 hospices, 5 of which are run by Christian organizations. During the first round of data collection in February 2022, 6 explicit statements on the websites of palliative care units (all of them were Christian) and 3 explicit statements on the websites of hospices (1 publicly funded and 2 funded by Christian organizations) were found. In August 2022, i.e. 6 months later this had hardly changed and only 1 additional statement of a publicly funded palliative care unit was added (see Table 3).

**Table 3:** Number of explicit statements of web-based publications on AS

	Number of organizations/units	Explicit statements on the assisted suicide Sterbeverfügungsgesetz in February 2022	Additional explicit statements on the assisted suicide Sterbeverfügungsgesetz in August 2022
*Palliative care units*	*43*	*6*	*1*
Public	34	0	1
Christian	9	6	–
*Hospice*	*14*	*3*	*–*
Public	9	1	–
Christian	5	2	–
*TOTAL*	*57*	*9*	*1*

In February 2022, indirect information could be found on one publicly funded palliative care unit and on two palliative care units funded by Christian organizations (both of which had also published an explicit statement). In August 2022, 2 additional indirect statements could be found, 1 from a public palliative care unit and 1 from a public hospice (see Table 4).

**Table 4:** Number of indirect information of web-based publications on AS

	Number of organizations/units	Indirect information on the assisted suicide Sterbeverfügungsgesetz in February 2022	Additional indirect information on the assisted suicide Sterbeverfügungsgesetz in August 2022
*Palliative care units*	*43*	*3*	*1*
Public	34	1	1
Christian	9	2	–
*Hospice*	*14*	*0*	*1*
Public	9	0	1
Christian	5	0	–
*TOTAL*	*57*	*3*	*2*

In summary, 9 explicit statements were found in February 2022, and only 1 explicit statement was added in August 2022, giving a total of 10 explicit statements 8 months after the AS law was introduced. Another 5 additional indirect forms of information were found (3 in February 2022 and 2 in August 2022).

Of all 57 palliative care and hospice institutions (43 palliative care units, 14 hospices), only 11 stated a position on AS on their websites. As 2 official statements were used by 3 institutions each, 13 different statements were analyzed in total (6 direct and 7 indirect; see Supplementary Table 5).

An analysis of the statements revealed the following three themes that are presented in narrative form using quotations from web-based publications to explore each theme in depth. The statements are marked with Sx (direct statement number “x”) or Ix (indirect statement number “x”). An asterisk (Sx*) is added in cases where the hospital/hospice is run by a Christian organization.

This qualitative study of Austrian hospices and palliative care institutions’ web-based statements about AS shows that only a few institutions shared statements. These covered three main themes: 1) demarcation: denial of involvement and judgment of AS, 2) duty: handling of requests and describing the target group of care recipients, and 3) explanation: experience, values, concerns, and demands.

#### Theme 1: demarcation

All statements denied any form of involvement in AS. They mentioned that the hospice or palliative care unit was no place for such action and nor would they provide help in carrying it out. Some statements were very short and clear, such as “Inpatient accompaniment of an assisted suicide is not carried out on the palliative care ward” (S6). Others added an explanation, such as “We reject any form of participation in assisted suicide. (…) The mission statement of our hospital from 2018 says as follows: ‘It is part of our earthly life that it comes to an end—but we do not actively participate in it’” (S2*). Another statement specified that “Our employees are not allowed to take any actions that help in a suicide. (…) We would like to point out that our facility is not a place for assisted suicide” (S1*).

This demarcation and the statements of the palliative institutions were often accompanied by a judgment of AS. The statements devalued and interpreted AS. An example of such devaluation is the statement that “If, despite our best efforts, a person living or being cared for in one of our facilities decides to take the step of AS, we convey that *we do not condone* and will not support it” (S1*, emphasis by the authors of this paper). Another statement mentioned that AS is not regarded as “dignified dying” (S2*).

Judgment was also obvious in the interpretation of requests for AS. Statements repeatedly mentioned requests as a “cry for help”; for example, “The wish to kill is often a cry for help and an expression of existential suffering” (I2*) and “Perhaps it is a call for help for attention?” (S4*). One palliative care unit deemed the demand for AS as being equal to experiencing oneself as a burden and stated: “We will in no way participate in allowing people who feel like a burden to disappear from our society” (S5*); however, one institution underlined in their statement that they discussed such requests openly and without judgment (S2*).

Judgment is also obvious in the choice of words and imagery. In general discourse, neutral expressions are mostly used to avoid stigmatization, such as “Assistierter Suizid” or “Assistierte Selbsttötung” in German; however, one palliative care unit used the German expression “Assistierter Selbst*mord*” (S5*, emphasis by the authors of this paper), which has a connotation of murder and illegality. Furthermore, this statement did not differentiate between AS and killing on demand.

The pictorial language added another layer of judgment. The websites showed a photo of a candle in pure darkness that was not illuminated by the light (S1*); a photo of wilted, dreary sunflowers with their heads hanging (I2*) and a mural of a Christian person with the text “God may not hear you according to your will, but He hears you for your salvation” (S2*), a sentence ascribed to the theologian Saint Augustine of Hippo (354–430 AD). Thus, the imagery added to the judgement that was expressed in the text.

#### *Theme 2: duty*

In taking a position regarding requests for AS, the palliative care units and hospices often shared in their statements how they planned to handle these requests in practice, what they saw as their duty, and the target group they cared for. Overall, the statements on AS also included mission statements, and the institutions claimed their engagement in caring for, accompanying, and treating people who need support and in offering conversation, empathetic attention, symptom relief, dignified living, and the best possible quality of life. This included minimizing fear and reducing suffering (see S1*, S2*, I4*). One palliative care unit emphasized that “Palliative care (…) would also like to accompany patients who, in their distress, express the wish to ‘want to die’” (S6).

Concerning how the institutions wanted to react to the demand for AS, several mentioned repeatedly that they would “take it seriously” (see S1*, S2*, S5*, I2*). This was further explained with the wish to understand and the aim to explore reasons and circumstances. They hoped to provide an open, value-free discussion and to give space. Additionally, it was stated that together with the individual, they planned to look for alternatives, provide consolation and relief, and provide care through different approaches and with the help of an interprofessional team (S1*, S5*). The statements also highlighted accepting the right to free self-determination and the will of the patient/resident (S1*, S2*, S4*, S5*). There was only one exception, which stated “Human dignity lies solely in one’s existence—and is not dependent on self-determination and meaning” (S4*).

The statements further emphasized several times that the institutions were “open for everyone” (S2*) but highlighted that they especially focused on “people who need care, treatment or support for their lives” (S1*) or on “particularly vulnerable people” (S2*). It was repeatedly mentioned that nobody should be left alone (S1*, I3*, I4*). The statements also mentioned that the institutions offered help and support for relatives, friends, and loved ones of the patients and the employees (S1*, S2*).

#### Theme 3: explanation

The web-based publications not only stated the institutions’ approach to requests for AS but also explained their line of action and point of view. These explanations can be attributed to three categories: experience, values, and concerns and demands.

Experience in earlier work was repeatedly stated as a reason for the institutions’ approach. This was stated in many ways. One palliative care unit said on its homepage that “Experience shows that if the pain is relieved and if the person is accepted and accompanied, then assisted suicide is not an issue” (S5*). In a similar vein, a hospice employee’s statement was cited on the hospice website, as follows: “My conviction and my experience from many, many conversations with hospice patients is that the desire for euthanasia falls silent when you say: ‘We are here for you’” (S4*). A Tirolean palliative care unit that is linked to a hospice emphasized symptom control with the statement “From our experience we know that the desire for active euthanasia is reduced as people experience effective pain relief, care, and solace” (I2*). Other institutions drew on their experience of observing the ambivalence of dying people and linked it to dignity, making statements such as “From our experience in caring for people, we know that there are different forms of wishes to die and that these are sometimes to be interpreted ambivalently. (…) Therefore, assisted suicide cannot be reconciled with our idea of a dignified death. This insight is based on our many years of experience in the treatment, care, and support of patients and their relatives” (S2*).

This connects to the next form of explanation, as the institutions often based their statements on values. An aspect that was mentioned many times was the importance of and emphasis on the protection of life (see S1*, S2*, S5*, I2*). In this respect, Christian beliefs and values were stressed. This can be seen in the following two examples: “In our Christian healthcare facilities, we respect the life of every human being as a gift from God. Because of the trust that Christ accompanies us to a fulfilled existence, the comprehensive protection of life is a matter close to our hearts” (S1*) and “For us as the Elisabethinen Order Hospital, it is also important in the future that the words of Franz Kardinal König remain the guiding principles not only for our institution but for society as a whole: ‘People should die at the hand of another human and not by the hand of another human’” (S2*).

The explanations also talked about the responsibility of the community, highlighting the embeddedness of everyone in broader structures. The following quotation exemplifies this: “Anyone who stylizes active euthanasia as a successful case of autonomy overlooks the existential social dimension of human beings: their fundamental dependence on others, their being integrated into the community. We are all connected, no one is an island in itself” (I2*).

The explanations for the statements included concerns and demands that the institutions had and shared. This ranged from financial concerns (no pressure on vulnerable people, possible impact of financial interests/incentives for institutions) to worries about the employees (shattering of the self-image of helping professions, the importance of freedom from coercion, and security for healthcare workers) and the often mentioned fear of the “slippery slope” and “Pandora’s box” (the idea that supply creates demand and the argument that AS could in the future be extended to seriously ill children or people with dementia, for example). This led to openly claimed demands, such as the protection of vulnerable groups, the prevention of abuse, mandatory clarification by a psychiatrist/psychologist, and comprehensive hospice and palliative care support in Austria, and the argument that such alternatives for seriously ill people would be the “most effective suicide prevention” (I5*).

These arguments led to the claim that a broader societal discussion was needed. In a published interview with a physician at a palliative care unit, the interviewee stated that “The new regulation of euthanasia is a major task for our society. (…) A social challenge” (I1*). Similar words were found on the webpage of another hospice: “[E]specially being dependent on one another is the humanistic expression of a society. As life-immanent realities, dying and death cannot be separated from people’s ability to relate socially” (S4*).

To summarize, the qualitative study of Austrian hospices’ and palliative care units’ web-based statements about AS showed that only a few institutions shared statements, and these covered the themes of demarcation, the institution’s duty, and their explanation of their position.

## Discussion

This qualitative study explored the positions taken by Austrian hospices and palliative care units online with respect to AS. The study results show that only very few institutions published a statement about AS on their website, and of the ones that did, the majority were Christian institutions. Up to August 2022, 8 months after AS was legalized in Austria, none of the publicly accessible online statements supported AS or showed an open attitude about it. Most statements were published in 2021, with only a few being added after AS became legal in January 2022, and there is a general lack of recent publications. The analysis showed that many motives for the statements could be found in the different institutions. As stated in other studies, religion plays a role in attitudes regarding AS. Several factors have been identified with respect to physicians’ opinions towards AS, with non-Catholic religion relating to a higher endorsement of AS [7–11].

It can be observed that many palliative medicine professionals view AS critically [7], even though it has also been shown that some support AS, such as 59% of hospice nurses who were asked in Oregon [12]. The British Medical Association carried out a survey on physician-assisted dying in 2021 that focused on the member’s opinions. Responses varied, depending on different medical specialties. For example, more than 60% of otolaryngologists, anesthesiologists, and emergency physicians endorsed physician-assisted suicide, while less than 30% opposed it. In addition, more than 75% of palliative care physicians opposed physician-assisted suicide, while fewer than 15% endorsed it [13].

The results of this study also suggest the reluctance of the Austrian palliative care community regarding AS. This might be due to several reasons. First, there is a lack of nationwide palliative and hospice care. A global analysis has shown that the level of palliative care provision has improved over the last years but has still not been fully integrated into the country’s health plan and does not meet the expected needs. Austria is on the same level as countries with a lower World Bank income level, such as the Czech Republic, Georgia, Kazakhstan, and Uganda [14]. Second, numerous concerns and suggestions that were expressed by palliative care organizations concerning the StVfG AS law in 2021 were barely taken into account in the final legislation. This also suggests that the short period in which the new law was formulated led to a lack of fundamental and in-depth discussions in Austria. The focus remained more on the concrete implementation.

More generally, AS poses a conflict between the values and the objective of a palliative healthcare professional’s work. According to the World Health Organization, palliative care “intends neither to hasten nor postpone death” [15]. Additionally, palliative care professionals mainly focus on ways to deal with existential suffering and try to deal with patients’ wishes to end their lives from a different perspective, as individuals often suffer from distressing symptoms that can be managed. In addition, palliative care focuses on advance care planning and shared decision making; patients can reject treatment (e.g., antibiotics in cases of infection), can voluntarily stop eating and drinking, or can consider palliative sedation in the case of refractory symptoms. Therefore, AS might not be seen as something necessary from the viewpoint of palliative care.

Regarding the continuation of care for terminally ill patients who do not wish for AS or to hasten death, a more complicated relationship may result when providing both palliative care and AS to patients. Patients and caregivers may not always be able to distinguish between AS and palliative care, and the purposes and goals of palliative care might lead to confusion, which has been shown in a qualitative study by Mathews et al. [16].

Many of the physicians currently working in palliative and hospice care are oriented towards traditional palliative care values. Of the physicians currently specializing in palliative care, 75% are employed but not self-employed in Austria [17]. This leads to an additional difficulty, as the purpose and mandate of hospitals are defined by the Hospitals and Health Resorts Act (Krankenanstalten und Kuranstaltengesetz, §1KAKuG), and hospitals are places where health status is determined and monitored through examination, prevention, amelioration, and the curing of illness with treatment.

Hospitals are therefore by definition facilities intended for the medical care of the chronically ill. Furthermore, they are places of treatment and not of residence, which is contrary to the legislator’s plan that AS should take place in an individual’s home and private space [1]. Physicians who are employed by institutions are therefore not free to decide whether they participate in AS within the facilities but are dependent on their employer’s and organization’s guidelines. As the law was introduced quickly, institutions had little time to develop guidelines, and hardly any guidelines are publicly available at the current time. In this respect, it should also be noted that when a healthcare facility offers AS, resources are consumed. This will lead to less time for other patients and contradicts the original assignment for hospitals to care for and treat patients. Institutions might also fear stigma once they openly state that AS is possible. It remains to be seen how the law develops. AS is generally intended to take place in a private space and not in healthcare institutions; however, patients will still want to have contact with their medical treatment team. Medical teams should be prepared for more questions about AS. It is also interesting to note that none of the web-based statements on AS included a disclaimer or helpline for suicide prevention.

Due to the timeliness of this study and the new legislation and the recent change of law in Austria, many questions remain open. The results of the current study show that patients who are looking for answers regarding AS via websites of hospices or palliative care units find very little information.

### Limitations/strengths

The current study’s limitations must be addressed. The findings are not generalizable beyond the new Austrian legislation. Therefore, the results are country-specific and not transferrable to other contexts. This topic requires further in-depth investigation. Websites are only one way of providing information, and as the Austrian StVfG on AS is new, the results of this study only provide a first overview. The novelty of this study should be emphasized as a strength, as the present work is the first to address the new legal situation in Austria from the perspective of the public positions of palliative and hospice facilities on AS.

## Conclusion

The results of the current study demonstrate how the changes in legislation are still making their way into the practices of Austrian hospices and palliative care units. It shows how few institutions have taken a position regarding the StVfG on AS and that the majority use their websites to state their opposition to AS or to state that their institution will not participate in AS. Austrian citizens who claim their right to AS and who may use the internet as their first source of information currently find little information. There is no online statement of a palliative care facility that supports AS, while negative attitudes from Christian institutions are predominant.

It is assumed that with time, patients will find more information via websites and that institutions will determine how to handle AS. Institutions will have to declare their position now that AS is legal under certain conditions and is an option for people with life-limiting illnesses in Austria.

### Supplementary Information

Table 5. References for statements
