# Supplementary material for: Statements of Austrian hospices and palliative care units after the implementation of the law on assisted suicide: A qualitative study of web-based publications
Source: Wien Klin Wochenschr. 2023 Mar 9;136(13-14):382–9. doi: 10.1007/s00508-023-02157-9 (PMC11239715; doi:10.1007/s00508-023-02157-9)
Supplement: Supplementary file 1 — Table 5. References for statements [file 508_2023_2157_MOESM1_ESM.docx]

**Supplement**

**Table 5**. References for statements

| Explicit statements | | | |
| --- | --- | --- | --- |
| S1* | 5.1.22  29.12.2021  not collectable | - Ordensklinikum Barmherzige Schwestern Linz  - Krankenhaus Barmherzige Schwestern Ried  - Göttlicher Heiland Wien | https://www.ordensklinikum.at/fileadmin/user_upload/1_images/2_Artikel/Wissenswertes/Positionspapier_Assistierter_Suizid.pdf  https://www.vinzenzgruppe.at/wir-ueber-uns/aktuelles/position-der-vinzenz-gruppe-zum-assistierten-suizid  https://www.khgh.at/ueber-uns/ethik |
| S2* | 1.6.21 | - Krankenhaus der Elisabethinen Graz  - Hospiz St. Elisabeth Graz  - VinziDorf Hospiz | https://www.elisabethinen.at/position-zum-assistierten-suizid/ |
| S3* | 15.11.21 | Palliativstation/Hospiz Hall | https://www.hospiz-tirol.at/tagebuch/2021/11/stellungnahme-des-dachverbandes-zum-entwurf-des-sterbeverfuegungsgesetzes/ |
| S4* | 14.09.21 | Albert Schweitzer Hospiz Graz | https://ggz.graz.at/de/Media/Dateien/PDF/Fachartikel/Fachartikel-Assistierter-Suizid-beim-alten-Menschen-Loesungsoption-oder-Oeffnen-der-Buechse-der-Pandora |
| S5* | not collectable | CS Hospiz Rennweg | https://www.cs.at/angebote/cs-hospiz-rennweg/am-lebensende |
| S6 | 03-07/22 | Hohenems | https://www.landeskrankenhaus.at/leistungsangebot/fuer-patienten/medizinische-fachbereiche/lkh-hohenems/palliativstation#editor1 |
| Indirect information | | | |
| I1* | 8.12.21 | Ordensklinikum Barmherzige Schwestern Linz | https://www.ordensklinikum.at/de/aktuelles/johann-zoidl-und-david-fuchs-im-ooen-interview-ueber-aengste-schmerzen-und-hoffnung-1336/ |
| I2* | 12.11.20 | Palliativstation/Hospiz Hall | https://www.hospiz-tirol.at/tagebuch/2020/11/aktive-sterbehilfe-werner-muehlboeck-nimmt-stellung/ |
| I3* | 1.11.21 | CS Hospiz Rennweg | https://www.derstandard.at/story/2000130802123/sterbehilfe-stellt-angehoerige-und-aerzte-vor-schwierige-fragen |
| I4* | 9.10.21 | CS Hospiz Rennweg | https://www.cs.at/presse/news-und-aktuelles/welthospiztag-9-10-2021 |
| I5* | 12/21 | CS Hospiz Rennweg | https://www.google.com/url?client=internal-element-cse&cx=010360184102331761898:ycctuumxzy0&q=https://www.cs.at/files/worte_zum_advent_weihbischof_kraetzl_2021_neu.pdf&sa=U&ved=2ahUKEwjZtKrzsfr1AhV3hP0HHTlFAfEQFnoECAEQAQ&usg=AOvVaw2zzvDxYgOHi-Y8SNsl_uw0 |
| I6* | 03/21 | CS Hospiz Rennweg | https://www.google.com/url?client=internal-element-cse&cx=010360184102331761898:ycctuumxzy0&q=https://www.cs.at/files/cs_cuspruch_3_2021_1.pdf&sa=U&ved=2ahUKEwjZtKrzsfr1AhV3hP0HHTlFAfEQFnoECAIQAQ&usg=AOvVaw3cmDhh7JVZcZF5O7KgEx9u |
| I7 | 2.6.21 | AKH Wien | https://innere-med-1.meduniwien.ac.at/palliativmedizin/news/single-view/?tx_ttnews%5Btt_news%5D=5174&cHash=cbafd16b8a900196f7a84761b54c30d3 |
